# Modelling of Chloride Transport in the Standard Migration Test including Electrode Processes

**DOI:** 10.3390/ma16186200

**Published:** 2023-09-14

**Authors:** Zine-Eddine Kribes, Rachid Cherif, Abdelkarim Aït-Mokhtar

**Affiliations:** LaSIE UMR CNRS 7356, La Rochelle University, 17000 La Rochelle, France; zine-eddine.kribes@univ-lr.fr

**Keywords:** chloride migration, thermodynamic equilibrium, multi-ion interactions, electrode processes

## Abstract

The modelling of chloride transport in concrete under an electrical field requires taking into account the electrode processes. These processes are very rarely introduced into the literature, despite their impact on chloride migration and the electroneutrality of the pore solution of the material. This paper aims to propose a multi-ion model for chloride migration that takes into consideration the electrode processes. The model is applied to simulate the standard chloride migration test. The generation of OH^−^ in the cathode and H^+^ in the anode allows for the monitoring of the electroneutrality. The model considers all of the ions in the pore solution. Ion fluxes are calculated using the Nernst–Planck equation. The Langmuir model is used to simulate the chloride isotherms. The thermodynamic equilibrium in the material is considered, which reflects the ion–solid interactions during the migration. Measurements of water porosity and the chemical composition of the pore solution are essential to provide input data and the initial and boundary conditions. The numerical results of the ion profiles in the material studied confirm the electroneutrality at any point within the material, in contrast with models that do not take the electrode processes into account. The proposed model allows for the more accurate simulation of the chloride migration test and electrochemical chloride extraction in reinforced concrete structures subjected to NaCl as part of maintenance and repair strategies.

## 1. Introduction

The corrosion of steel in reinforced concrete (RC) structures in coastal zones is mainly induced by chloride ingress, which presents a permanent risk of degradation. Chlorides penetrate into the cover concrete under a concentration gradient between seawater and the concrete pore solution and/or liquid pressure gradient in partially saturated concrete when heat and moisture transfers occur (tidal areas or marine fogs). When the chlorides reach the rebars at a threshold concentration, they generate rebar depassivation and corrosion [1,2,3]. Given the high cost of maintenance/repair, nowadays, the durability of RC structures in their environments is one of the main challenges mentioned in the specifications of construction.

In the last decades, several experimental studies were proposed to investigate the durability of reinforced concrete and its behavior in the face of environmental aggressions. Golewski [4] studied the impact of the incorporation of coal fly ash (CFA) on water movement within concrete. The study aimed to establish a relationship between water absorption and the compressive strength of concretes with varying substitution rates of fly ash (0%, 20%, and 30%). The results indicated that the incorporation of 20% CFA in the binder composition enhanced both the compressive strength and water absorption of the concrete. The microstructure of concrete containing 20% CFA displayed a porous and loosely structured nature, resulting in heightened water absorption. However, the addition of 30% CFA led to a significant decrease in both the compressive strength and water absorption compared to concrete without CFA. Yang et al. [5] showed the impact of stray currents on structures like subways that accelerate rebar corrosion in concrete. Rebar corrosion and calcium dissolution are affected by the concrete strength grade, rebar diameter, and stray current intensity. Additionally, the impact of rebar corrosion and calcium dissolution on concrete strength was studied via the simulation of a stray current environment and the execution of an electrified acceleration test. The results indicated that higher concrete strength grades correlated with lower stray current intensity, resulting in decreased corrosion and calcium dissolution. In this sense, Liu et al. [6] investigated the effect of stray current on calcium leaching and the compressive strength of cementitious materials. The study examined the impact of the stray current magnitude (20 V, 40 V, and 60 V), water-to-binder ratio (w/b = 0.40, 0.5, and 0.6), fly ash ratio (5%, 10%, and 15%), and silica fume ratio (5%, 10%, and 15%). A prolonged exposure to stray current increases the mass of the cathode-side leachables and decreases the compressive strength of the materials. After 120 d of stray current, the w/b of 0.50 exhibited a minimal reduction in compressive strength while the w/b of 0.40 showed the highest cathode-side leachables and the w/b of 0.60 showed the lowest ones. An increased fly ash content reduces cathode-side leachables. An amount of 10% silica fume leads to the lowest cathode-side leachables and the lowest compressive strength decrease.

More specifically, several experimental and numerical studies were developed to propose methods and tools for predicting chloride transport in cementitious materials. The standard migration test is used to determine the chloride diffusion coefficient in a steady state or non-steady state [7,8,9,10,11,12]. Recently, Szweda et al. [13] compared several standardized methods for determining the chloride diffusion coefficient in concrete using the thermodynamic model of migration. The authors highlighted a dispersion of the obtained results from the experimental methods compared with the thermodynamic model. Moreover, single-ion modelling was used to predict chloride transport in cementitious materials [14]. After that, many multi-ion approaches were developed that took into consideration several ions in the pore solution and more chemical and physical interactions during transport [15,16,17,18,19,20,21]. Xia and Li [22] proposed the numerical modelling of ion transport in saturated cementitious materials based on the Poisson–Nernst–Planck (PNP) equations, taking into consideration the chemical interactions between the monovalent ions in the pore solution in order to monitor the impact of the interactions on chloride ingress. Fenaux et al. [23] proposed a chloride transport model in saturated concrete taking into account the monovalent and divalent ions of the pore solution: Cl^−^, Na^+^, K^+^, OH^−^, and Ca^2+^. The diffusion, migration, and chemical activity were considered. The chemical activity coefficient was calculated using the Pitzer model. The numerical results highlighted the influence of the composition of the pore solution and chemical activity on chloride penetration.

Furthermore, recent research discussed the impact of the thermodynamic equilibria on chloride reactive transport in cementitious materials [24,25,26,27]. Yu and Zhang [28] proposed a model for predicting the leaching of cement paste in an ammonium nitrate solution taking into account the ion transport, chemical kinetics, and thermodynamic equilibria. The ion transport was calculated using the PNP equation, while the chemical activity was calculated using the Davies model. Tran et al. [29,30] developed a coupling between chloride transport and the thermodynamic equilibrium, taking into consideration the kinetic control to predict chloride fixation in concrete. Jensen et al. [31,32] developed a multi-ion approach for reactive mass transport in a saturated mortar exposed to chlorides for 180 days, including the chemical equilibria. Cherif et al. [33] proposed a coupling between multi-ion transport and thermodynamic equilibria in cementitious materials containing mineral additions. The model took into consideration all of the ions of the pore solution, the portlandite dissolution, and Friedel’s and Kugel’s salt precipitation during chloride transport. Ion fluxes were calculated using the Nernst–Planck equation, while the thermodynamic coupling was based on the low mass action and rate of dissolution/precipitation of the solid phases.

The models mentioned above are generally based on the PNP equation using a limited number of ions in the pore solution whose concentrations are significant. The other models consider all of the multi-ion interactions in the material, but they are based on Fick’s law, which misdescribes chloride ion transport. Researchers dealing with chloride migration with consideration for the electrode processes have very little literature.

Motivated by this need, the multi-ion modelling of chloride migration taking into consideration the electrode processes is proposed in this study. The model is applied to Portland cement-based concrete subjected to the standard chloride migration test. Experimental investigations on the total porosity and composition of the pore solution of the concrete are performed in order to feed the model in terms of input data and initial conditions. The electrode processes reflect the generation of OH^−^ and H^+^ in the cathode and anode, respectively. The electrode processes ensure the electroneutrality in the migration cell (sample and compartments). The concentrations of OH^−^ and H^+^ are calculated from the current density measured during the test using Faraday’s law. The charge passed is deduced from the current density measured. Ion fluxes are calculated using the Nernst–Planck equation, which describes the diffusion and migration of the ions. The Langmuir model is used to simulate the chloride chemical fixation by the material (chloride isotherms). The chemical activity is neglected according to Truc et al. [34]. The considered ions are Cl^−^, Na^+^, K^+^, OH^−^, H^+^, Ca^2+^, and SO_4_^2−^. The migration cell used is composed of two compartments: (1) upstream containing 25 mM NaOH, 83 KOH, and 500 mM NaCl; (2) downstream containing only 25 mM NaOH and 83 KOH (boundary conditions). The composition of the pore solution of the material tested is considered as the initial condition. An electrical field of 300 V∙m^−1^ is applied at the sample boundaries and monitored using two calomel reference electrodes. The latter were placed at each side of the sample tested in order to maintain the electrical field constant. The modelling outputs are as follows:

- The ion profiles in the material that can be used for the calculation of the ion diffusion coefficients in the non-steady state (from the ion-penetration depth), with and without the electrode processes.
- The electroneutrality in the sample tested with and without integrating the electrode processes in order to highlight the need for the consideration of the electrode processes in the chloride migration model proposed.

## 2. Experimental Program

### 2.1. Materials

For this study, specimens of concrete based on Ordinary Portland cement CEM I 52.5 N in compliance with European standard NF EN 197-1 were used. The manufacturer provided the mass fractions of the primary clinker phases based on: 65% C_3_S, 13% C_2_S, 7% C_3_A, 13% C_4_AF, and 4.9% gypsum. The chemical composition and physical properties (density and Blaine specific surface) of the cement are given in Table 1.

**Figure 1:**
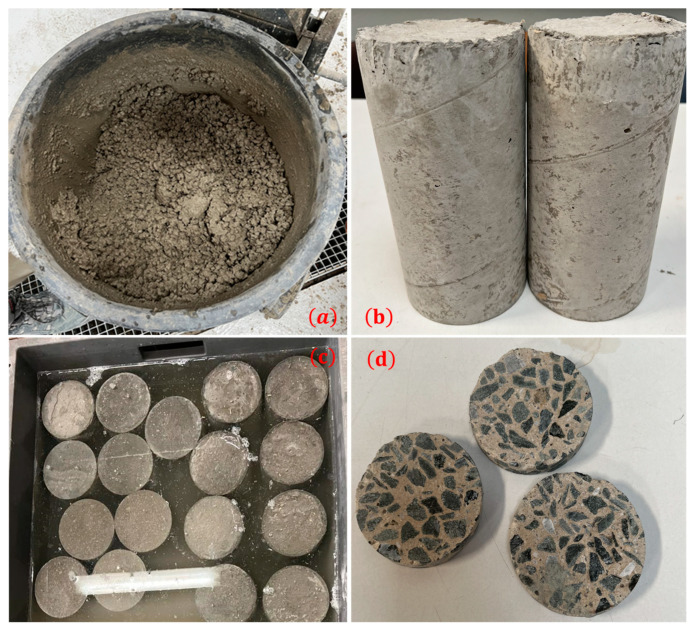
Ordinary concrete used: (**a**) during mixing; (**b**) after demolding; (**c**) during curing; and (**d**) test samples used.

Cylindrical concrete specimens of 11 cm in diameter and 22 cm in height were cast and demolded 24 h after manufacturing. The composition of 1 m^3^ of the ordinary concrete used was: 300 kg of cement, 710 kg of sand, 1242 kg of coarse aggregate, and 180 kg of water. Then, the specimens were stored in a Ca(OH)_2_-saturated solution for 1 year. After that, three samples were cored from the center of these specimens in order to avoid wall effects and heterogeneity. The samples were used to measure the porosity. Figure 1 shows photos of the ordinary concrete used and samples tested.

### 2.2. Tests and Procedure

**Figure 2:**
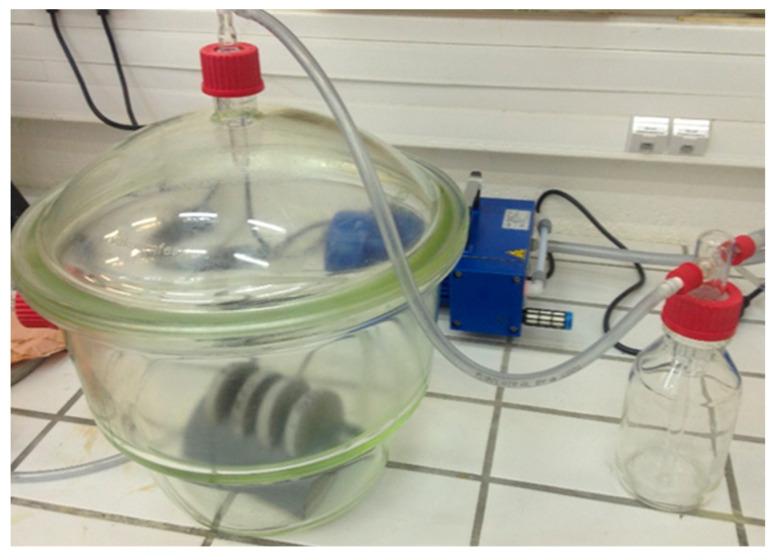
Desiccator and pump for water porosimetry.

The water porosity (*φ*) was measured as per the French standard NF P 18-459 [35]. Using a desiccator, the material tested was first vacuumed to remove air bubbles and then soaked in water (see Figure 2). After immersion, we measured the mass of the saturated material (*M_s_*) and its mass in water (*M_w_*) using hydrostatic weighing. Then, the materials were dried at 45 °C and weighed to determine their dry mass (*M_d_*). Finally, the water porosity was calculated using Equation (1):

(1) $$\varphi \;\left[\%\right]=\frac{M_{s}-M_{d}}{M_{s}-M_{w}}\times 100$$

**Figure 3:**
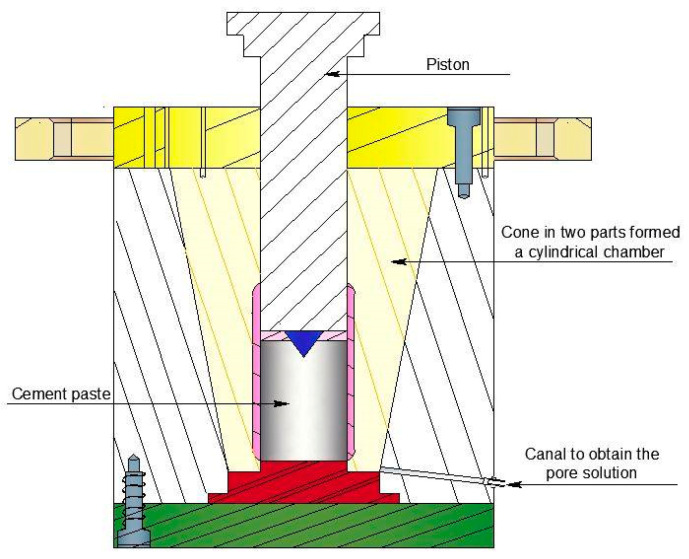
Schematic view of the OpiCAD^®^ extraction device used.

The pore solution was extracted using pressure as per the method outlined by Longuet et al. [36] and Barneyback and Diamond [37]. As the extraction of the pore solution from the concrete using this method was very difficult, giving a small and unrepresentative quantity of solution, we extracted the pore solution from a cement paste equivalent to the concrete studied. We proceeded with the assumption that the effect of aggregates on the composition of the pore solution was negligible, i.e., the aggregates in the concrete were inert and did not intervene in the chemical composition of the pore solution. The extraction press used is composed of a cylindrical chamber and a hardened steel piston as shown in Figure 3. A cylindrical sample of 5 cm diameter and 7 cm height is placed in the chamber under the piston that is submitted to step loading using a hydraulic press in order to ensure a controlled and gradual extraction process [8]. A loading of 0.02 MPa·s^−1^ was applied for 15 min followed by an idle phase for 5 min. Finally, an average of 8 mL of pore solution was collected. After that, the pore solution was stored in a refrigerator at 3 °C for at least 24 h. Then, the solution was analyzed using ion chromatography.

## 3. Methodology

### 3.1. Modelling Principle

The time evolution of the ion concentration (*C_i_*) during the migration test is calculated using the mass balance equation (see Equation (2)), which takes into account the porosity of the material tested (*φ*), the chloride concentration bonded to the cement matrix (*C_i,b_*) calculated using the Langmuir’s model, and the ion flux (*J_i_*) calculated using the NP equation (see Equation (3)). The internal electrical potential between ions is neglected in front of the applied electrical field of 300 V.m^−1^. The mass exchange term (*q_i_*), added to the mass balance equation, describes the ion gain/loss in the pore solution due to the dissolution/precipitation of the solid phases considered (C-S-H, portlandite, monosulfoaluminates, and trisulfoaluminates). Further details about the calculations of the term (*q_i_*) and the thermodynamic equilibrium constants used are shown in Table 1 and Table 2 of Cherif et al. [33]. The ions considered in this study are: Cl^−^, Na^+^, K^+^, OH^−^, H^+^, Ca^2+^, and SO_4_^2−^. Note that the proposed model is concerned with the ion transport in saturated materials that do not require coupling with convection and moisture transfer.

(2) $$\frac{\partial C_{i}}{\partial t}+\frac{\left(\left(1-\varphi \right)C_{i,b}\right)}{\partial t}=-div\left(J_{i}\right)-\frac{\partial q_{i}}{\partial t}$$

(3) $$J_{i}=-D_{E,i}\left(\nabla C_{i}+\frac{z_{i}C_{i}FE}{RT}+C_{i}\nabla ln\gamma_{i}\right)$$

where *D_E,i_* [m²∙s^−1^] is the effective diffusion coefficient of the ion *i*, *z_i_* is the valence of the ion *i*, *F* [C∙mol^–1^] is the Faraday constant, *E* [V∙m^−1^] is the applied electrical field, *R* [J∙K^−1^∙mol^−1^] is the ideal gas constant, *T* [K] is the temperature, and *γ_i_* is the ion activity coefficient.

The electrode processes responsible for the generation of OH^−^ in the catholyte (upstream) and H^+^ in the anolyte (downstream) are given in the following formulas. Note that non-corrodible Platine electrodes were used.

Cathode: 2 H_2_O + 2 e^–^ → H_2_ + 2 OH^–^ (4)

Anode: H_2_O → 0.5 O_2_ + 2 H^+^ + 2 e^–^ (5)

### 3.2. Case Study

**Figure 4:**
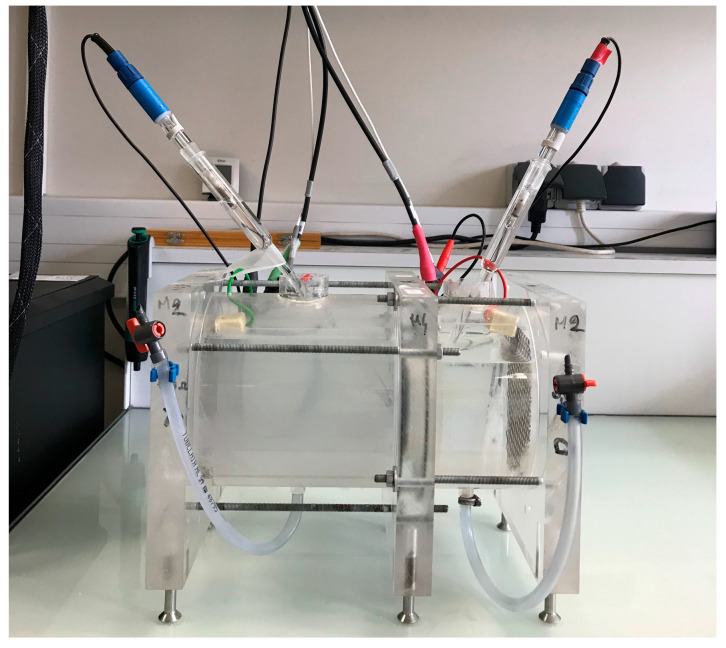
The chloride standard migration test used.

The experimental investigation involved filling the model with input data such as the water porosity and the chemical composition of the pore solution. The proposed model is applied to an ordinary concrete subjected to a 15-day standard migration test. The model allows for the simulation of all of the ion movements in the migration cell during the standard chloride migration test in the steady state. The standard migration test simulated is composed of two compartments separated by the test sample: the upstream and downstream compartments containing a basic solution of 25 mM NaOH and 83 KOH. In addition, 500 mM of NaCl is added to the upstream compartment. An electrical field of 300 V∙m^−1^ is applied at the sample boundaries. Concrete samples of 1 cm thickness are used (1D modelling). The standard migration test used is shown in Figure 4.

The mathematical model used was implemented using the COMSOL Multiphysics^®^ 5.5 numerical simulation environment based on the finite element method. This software is particularly well suited to multiphysics problems where several phenomena are studied simultaneously. The general form of partial differential equations was used to describe the multi-ion transport model proposed. The boundary conditions are given in Table 2. In this study, the numerical results show the ion profiles in the material tested that are useful for the calculation of the diffusion coefficient in the non-steady state [38,39].

## 4. Results and Discussion

### 4.1. The Water Porosity and Chemical Composition of the Pore Solution

Table 3 shows the water porosity and chemical composition of the paste pore solution equivalent to the concrete used. The results are in accordance with those of Andersson et al. [40]. Further details about the pore solution of the materials used are given in Cherif et al. [8].

### 4.2. Numerical Results

**Figure 5:**
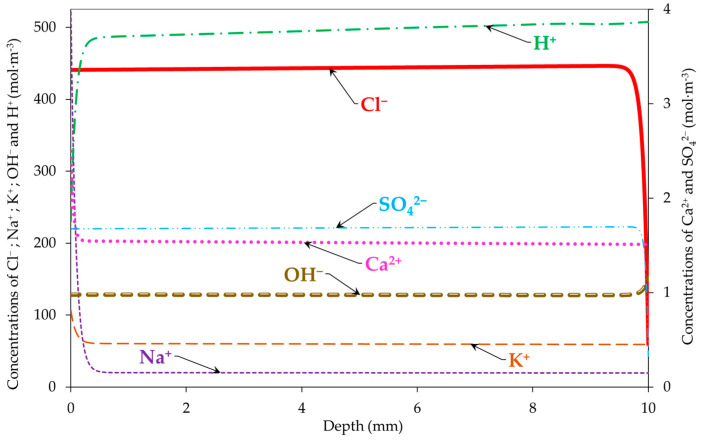
Profiles of Cl^−^, Na^+^, K^+^, OH^−^, H^+^, Ca^2+^, and SO_4_^2−^ in the sample at the end of the migration test (15 days) taking into consideration electrode processes.

**Figure 6:**
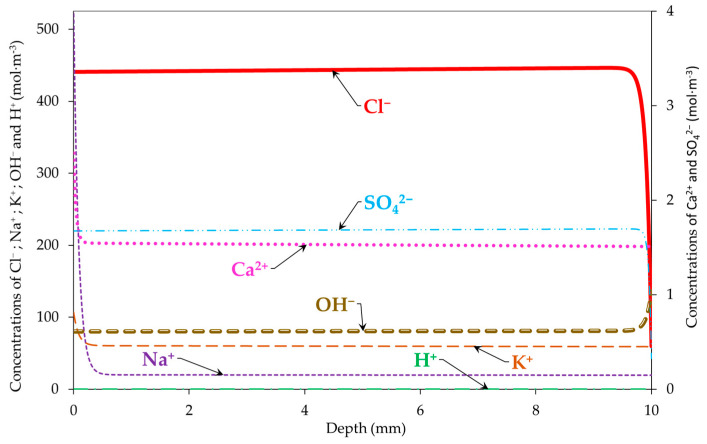
Profiles of Cl^−^, Na^+^, K^+^, OH^−^, H^+^, Ca^2+^, and SO_4_^2−^ in the sample at the end of the migration test (15 days) without taking into consideration electrode processes.

Figure 5 and Figure 6 show the simulated profiles of Cl^−^, Na^+^, K^+^, OH^−^, H^+^, Ca^2+^, and SO_4_^2−^ in the sample at the end of the migration test (after 15 days), with and without taking into consideration the electrode processes, respectively. The free chloride concentration in the pore solution is maximal along the sample depth (~440 mol∙m^−3^) because of the migration from the upstream compartment to the sample. The maximum concentration is relatively different compared with that in the literature’s data without taking into consideration the thermodynamic equilibrium (the participation of chlorides with the other ions in the pore solution to form salts). For the models in the literature, the maximum concentration is equal to the boundary condition on the side of the upstream compartment (500 mol∙m^−3^) [14].

Moreover, we noted an increase in the concentrations of Na^+^ and K^+^ in the pore solution due to their migration from the downstream compartment to the sample tested. The increases in Ca^2+^ and SO_4_^2−^ concentrations are due to the dissolution of the portlandite, monosulfoaluminates, and trisulfoaluminates under the electrical field [41,42].

**Figure 7:**
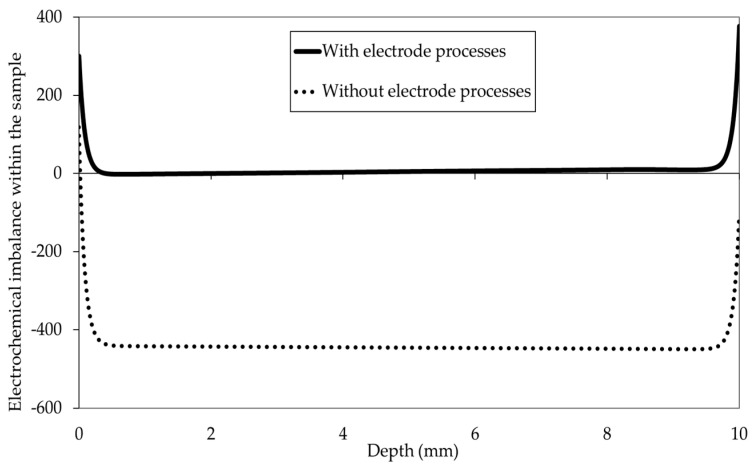
Electrochemical imbalance in the pore solution of the sample, deduced from ion concentrations in the steady state with and without taking into consideration electrode processes.

Finally, a difference between the concentrations of H^+^ and OH^−^ with and without taking into consideration the electrode processes is noticed. This is reflected by the electroneutrality ensured in the case of migration modelling with the electrode processes and not ensured in the case of modelling without the electrode processes (see Figure 7). This also impacts the concentration of the other ions in the pore solution. The electroneutrality was calculated using Equation (6). The results obtained confirm the need for taking into consideration the electrode processes in chloride migration modelling.

(6) $$\sum C_{i}z_{i}=0$$

## 5. Conclusions

From the obtained results, the following main conclusions are made:

- The proposed model allows for the simulation of the standard migration test in the steady and non-steady states taking into consideration the real pore solution of the material tested and the dissolution/precipitation phenomena during the migration. The modelling was applied to OPC-based materials.
- The outputs of the proposed model are the ion profiles of the material tested during the migration test.
- The numerical results show the need to take into consideration the electrode processes in chloride migration modelling in order to better simulate the standard migration test. The proposed model can be applied to any material whose porosity and pore solution composition are known.
- The proposed model could be improved by taking into consideration more solid phases of the material such as C-S-H, oxychloride, etc. for thermodynamic equilibrium coupling. Furthermore, it would be interesting to couple the proposed multi-ion diffusion model with moisture and heat transfer for application to the prediction of the durability of reinforced concrete structures exposed to chlorides in tidal zones.

## Figures and Tables

**Table 1:** The chemical composition and physical properties of the cement used.

Composition	CaO	SiO_2_	Al_2_O_3_	Fe_2_O_3_	SO_3_	K_2_O	Na_2_O	Chlorides	Density	Blaine Finesses (m²/kg)
CEM I (wt; %)	64.20	20.50	5.00	3.90	2.5	0.29	0.05	1.4	3.80	405

**Table 2:** The boundary conditions used.

	Boundary Conditions	
	Upstream (x = 0, t) [mol∙m^−3^]	Downstream (x = L, t) [mol∙m^−3^]
C_Cl_^−^	500	0
C_Na_^+^	525	25
C_K_^+^	83	83
C_OH_^−^	108	108
C_Ca_^2+^	0	0
C_SO4_^2−^	0	0

**Table 3:** The water porosity and chemical composition of the pore solution of the paste equivalent to the concrete used.

	Concrete (t = 0, 0 < x < L)
φ [%]	12
C_Cl_^−^ [mol∙m^−3^]	4
C_Na_^+^ [mol∙m^−3^]	51
C_K_^+^ [mol∙m^−3^]	117
C_OH_^−^ [mol∙m^−3^]	164
C_Ca_^2+^ [mol∙m^−3^]	2
C_SO4_^2−^ [mol∙m^−3^]	2

## Data Availability

Data are available from the authors on request.
